# 22. An Innovative Approach to Examining the Waning of Vaccine Effectiveness Using Automated Healthcare Data

**DOI:** 10.1093/ofid/ofab466.224

**Published:** 2021-12-04

**Authors:** Laurie Aukes, Joan Bartlett, Bruce Fireman, John Hansen, Ned Lewis, Morgan Marks, Patricia Saddier, Nicola P Klein

**Affiliations:** 1 Kaiser Permanente Vaccine Study Center, Oakland, California; 2 Kaiser Permanente Vaccine Study Center, Oakland, California, Oakland, California; 3 Kaiser Permanente Northern California, Oakland, California; 4 Merck and Co Inc., North Wales, PA; 5 Merck & Co., Inc., Kenilworth, New Jersey

## Abstract

**Background:**

We conducted a large real-world study of the long-term vaccine effectiveness (VE) of the live attenuated zoster vaccine (Zostavax; ZVL). Using an innovative approach with automated observational data we measured VE for incident herpes zoster (HZ) and severe HZ outcomes including post-herpetic neuralgia (PHN), herpes zoster ophthalmicus (HZO), and hospitalized HZ. This approach could be useful in long-term effectiveness studies of other vaccines.

**Methods:**

We assessed VE against HZ, PHN, HZO and hospitalized HZ for up to 10+ years after vaccination at Kaiser Permanente Northern California. We identified incident cases using diagnoses, laboratory tests and prescriptions, and validated a sample by chart review. For each outcome, we used a Cox regression model with a calendar timeline to estimate VE in relation to year since vaccination. The model for HZ included 11 time-varying vaccination status indicators to denote -- at each timepoint during follow-up -- either the number of years since ZVL vaccination (30 days to < 1 year, 1 to < 2 years, . . ., and 10+ years) or that the individual is unvaccinated (reference group). Analyses were adjusted for demographics and time-varying measures of immune compromise status, healthcare use and comorbidities.

**Results:**

From 2007-2018, 1.5 million people contributed to analyses; 507,000 (34%) were vaccinated. During 9 million person-years of follow-up, we observed 75,135 HZ cases, including 4,982 (7%) with PHN, 4,418 (6%) with HZO, and 555 (< 1%) who were hospitalized. VE for HZ was 67% (95% Confidence Interval [CI]: 65-69%) in the first year after vaccination, waned to 50% (CI: 47-52%) in the second year after vaccination, and then waned more gradually to 15% (CI: 5-24%) by 10+ years after vaccination. Initial VE was higher against PHN (83%; CI: 78-87%) and hospitalized HZ (89%; CI: 67-97%) with less waning observed over time (42% by Year 8 for PHN and 53% in Years 5 to < 8 for hospitalized HZ). VE against HZO was 71% in Year 1 and waned to 29% in Years 5 to < 8.

**Conclusion:**

Our large population, long follow-up and innovative methods let us estimate VE against HZ, PHN, HZO and hospitalized HZ for 10+ years after vaccination. Our approach could help assess waning and need for boosters for vaccines against other agents including COVID-19.

**Disclosures:**

**Morgan Marks, PhD, ScM**, **Merck & Co Inc.** (Employee) **Patricia Saddier, MD, PhD**, **Merck & Co Inc** (Employee) **Nicola P. Klein, MD, PhD**, **GlaxoSmithKline** (Grant/Research Support)**MedImmune** (Grant/Research Support)**Merck & Co Inc** (Grant/Research Support)**Pfizer** (Grant/Research Support)**Protein Sciences (now Sanofi Pasteur**) (Grant/Research Support)**Sanofi Pasteur** (Grant/Research Support)

